# Direct Comparisons of Illumina vs. Roche 454 Sequencing Technologies on the Same Microbial Community DNA Sample

**DOI:** 10.1371/journal.pone.0030087

**Published:** 2012-02-10

**Authors:** Chengwei Luo, Despina Tsementzi, Nikos Kyrpides, Timothy Read, Konstantinos T. Konstantinidis

**Affiliations:** 1 School of Biology and Center for Bioinformatics and Computational Genomics, Georgia Institute of Technology, Atlanta, Georgia, United States of America; 2 School of Civil and Environmental Engineering, Georgia Institute of Technology, Atlanta, Georgia, United States of America; 3 Department of Energy (DOE) Joint Genome Institute, Walnut Creek, California, United States of America; 4 Department of Human Genetics, Emory University, Atlanta, Georgia, United States of America; Universidad Miguel Hernandez, Spain

## Abstract

Next-generation sequencing (NGS) is commonly used in metagenomic studies of complex microbial communities but whether or not different NGS platforms recover the same diversity from a sample and their assembled sequences are of comparable quality remain unclear. We compared the two most frequently used platforms, the Roche 454 FLX Titanium and the Illumina Genome Analyzer (GA) II, on the same DNA sample obtained from a complex freshwater planktonic community. Despite the substantial differences in read length and sequencing protocols, the platforms provided a comparable view of the community sampled. For instance, derived assemblies overlapped in ∼90% of their total sequences and *in situ* abundances of genes and genotypes (estimated based on sequence coverage) correlated highly between the two platforms (R^2^>0.9). Evaluation of base-call error, frameshift frequency, and contig length suggested that Illumina offered equivalent, if not better, assemblies than Roche 454. The results from metagenomic samples were further validated against DNA samples of eighteen isolate genomes, which showed a range of genome sizes and G+C% content. We also provide quantitative estimates of the errors in gene and contig sequences assembled from datasets characterized by different levels of complexity and G+C% content. For instance, we noted that homopolymer-associated, single-base errors affected ∼1% of the protein sequences recovered in Illumina contigs of 10× coverage and 50% G+C; this frequency increased to ∼3% when non-homopolymer errors were also considered. Collectively, our results should serve as a useful practical guide for choosing proper sampling strategies and data possessing protocols for future metagenomic studies.

## Introduction

From the human gastrointestinal tract to the ocean abyss, whole-genome shotgun metagenomics is revolutionizing our understanding of the structure, diversity, and function of microbial communities [1], [2], [3], [4]. Next generation sequencing (NGS) technologies, such as the Roche 454, Illumina/Solexa, and, to a lesser extent, ABI SOLiD, have been cornerstones in this revolution [5], [6], [7]. For example, the high coverage of indigenous communities provided by NGS has made it possible to quantitatively assess the impact of diet on human gut microbiota [8] and the diversity of metabolic pathways within marine planktonic communities [9]. NGS platforms produce millions of short sequence reads, which vary in length from tens of base pairs (bp) to ∼800 bp. Even though read lengths increase as the technologies advance, they are still far shorter than the desirable length (e.g., the average bacterial gene length is ∼950 bp) or the read length obtained from traditional Sanger sequencing (∼1000 bp). Therefore, a desirable, first step in the analysis of metagenomic data frequently is to assemble sequences into longer contigs and, ultimately, into complete genome sequences. Analyzing raw (not assembled) reads, as opposed to assembled contigs, is typically restricted to cases where community complexity is too high or to specialized studies that aim to determine *in situ* abundance and/or population genetic structure and recombination [4], [10].

It is critical to assess the quality of the derived assemblies; to this end, several studies have recently attempted to evaluate the sequencing errors and artifacts specific to each NGS platform. For instance, it has been established that Roche 454 has a high error rate in homopolymer regions (i.e., three or more consecutive identical DNA bases) caused by accumulated light intensity variance [5], [11] and up to 15% of the resulting sequences are often products of artificial (*in vitro*) amplification [12]. Illumina does not appear to share these limitations but it has its own systematic base calling biases [13]. Most importantly, different tiles of the sequencing plate tend to produce reads of different quality [14], the 3′ ends of sequences tend to have higher sequencing error rates compared to the 5′ ends [15], and increased single-base errors have been observed in association with GGC motifs [16]. Algorithms that detect and correct these errors are being developed and incorporated into existing data processing pipelines.

It should be noted, however, that most of the previous error estimates and sequencing biases have been determined based on relatively simple DNA samples (e.g., a single viral genome) and thus, their relevance for complex community DNA samples remains to be evaluated. More importantly, it is currently unclear how the above limitations affect the quality of the gene and genome sequences assembled from complex DNA samples, and whether the technologies provide different estimates of the genetic diversity in a sample due to their inherent chemistry and protocol differences. To provide new insights into these issues, we evaluated the two most frequently used platforms for microbial community metagenomic analysis, the Roche 454 FLX Titanium and the Illumina GA II, by comparing and contrasting reads and assemblies obtained from the same community DNA sample.

## Results

### Genetic diversity recovered in raw (not assembled) reads and assembled contigs

**Figure 1:**
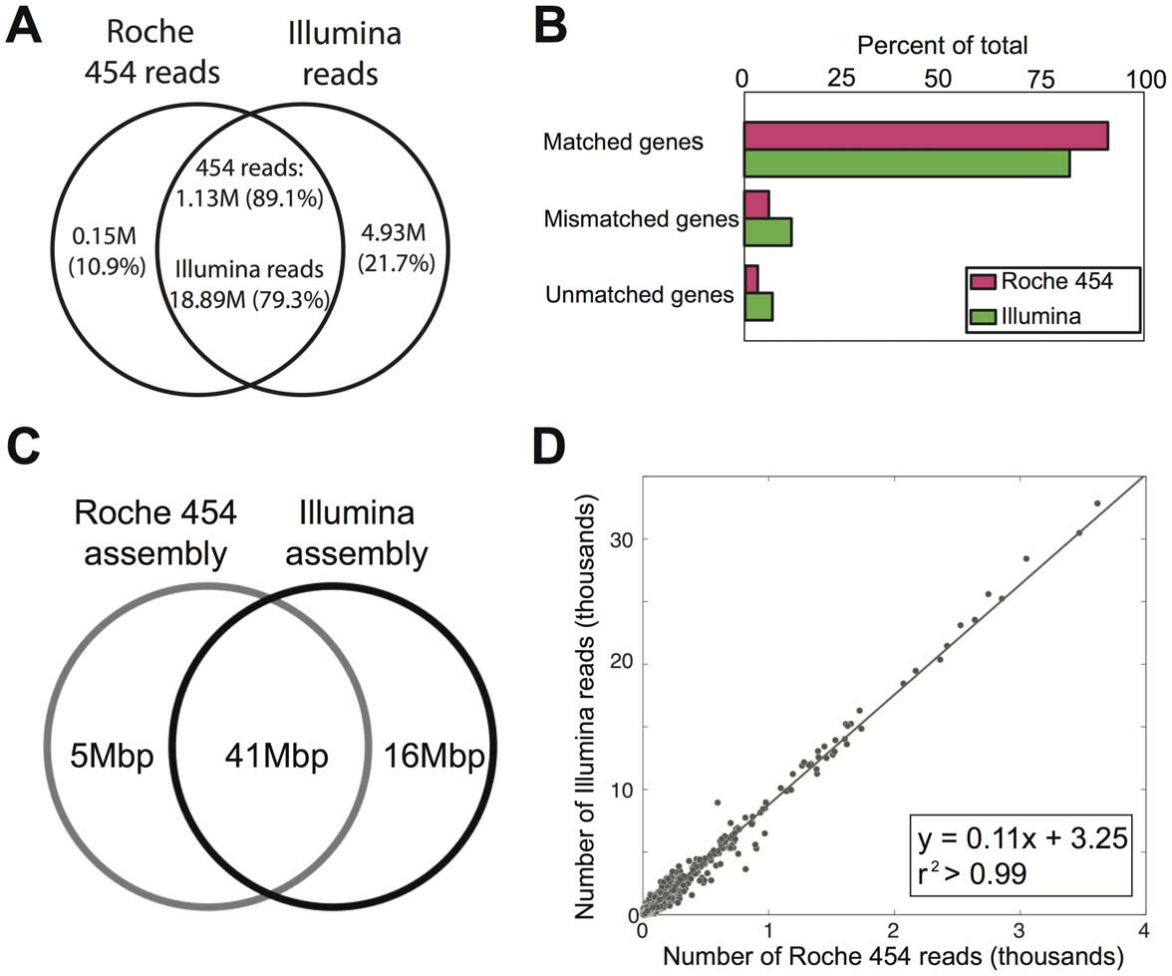
Genetic diversity and gene abundance in Roche 454 vs. Illumina data. (**A**) Venn diagram showing the extent of overlapping and platform-specific raw reads between the Lanier.454 and Lanier.Illumina datasets (without assembly). (**B**) Protein sequences annotated on raw (not assembled) reads matched genes in the reference assembly more frequently for the Roche 454 than the Illumina data. Conversely, protein sequences annotated on Illumina reads more frequently matched to the wrong protein sequence in the reference assembly (mismatched genes) or did not match any reference gene (unmatched genes). (**C**) Assemblies were obtained from 502 Mbp of Roche 454 and 2,460 Mbp of Illumina data using established protocols. Venn diagram showing the extent of overlapping and platform-specific sequences of assembled contigs longer than 500 bp. (**D**) Number of Roche 454 (x-axis) and Illumina (y-axis) reads mapping on the same contig shared between the two assemblies.

**Figure 2:**
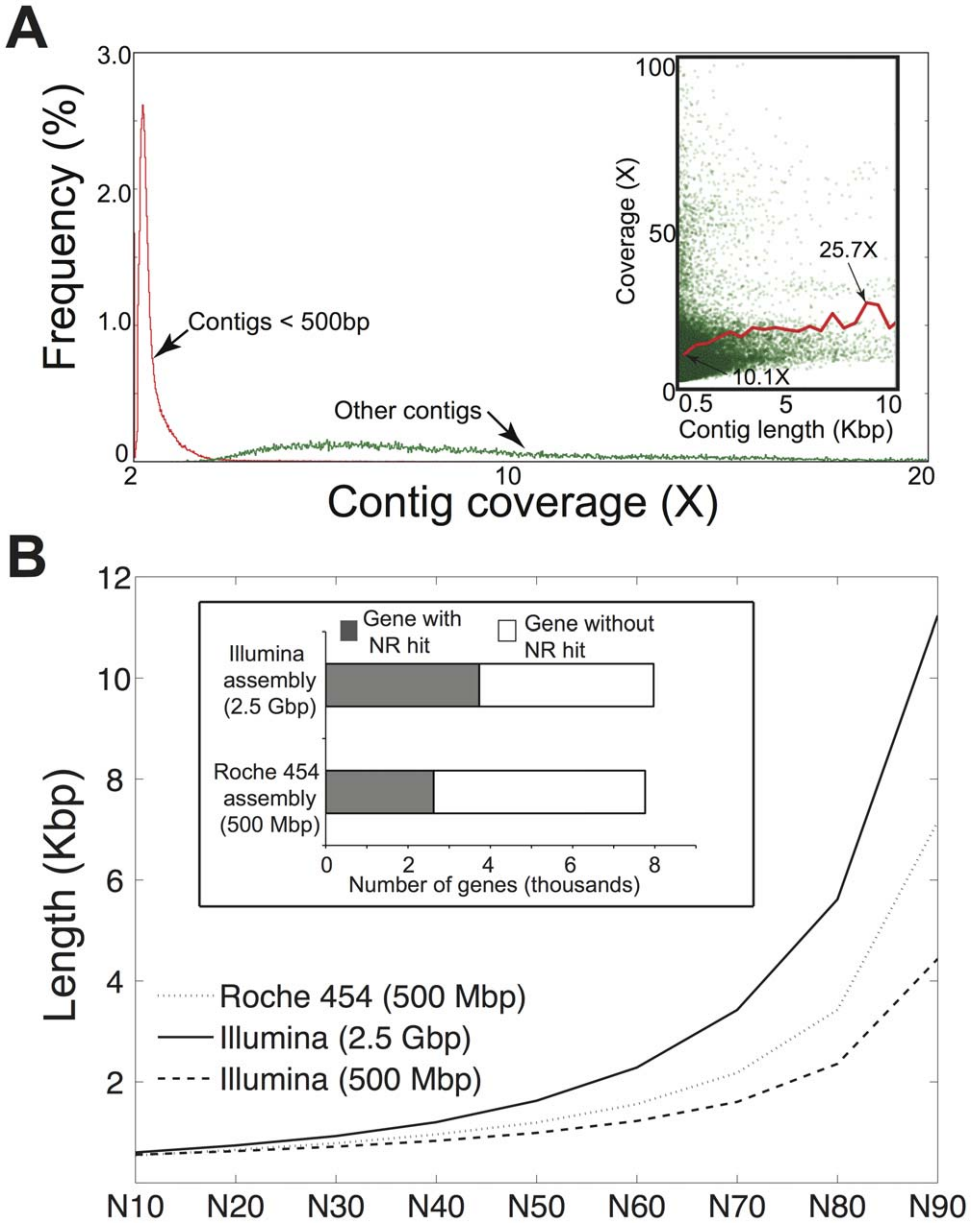
Average length and sequence accuracy comparisons of the Roche 454 and Illumina assembled contigs. (**A**) Length and coverage distribution of the contigs assembled from the Lanier.Illumina dataset. Note that contigs shorter than 500 bp (red) were numerically more abundant than longer contigs (green) but were characterized by substantially lower coverage (inset). (**B**) Graph shows the comparison of the contig length of three assemblies plotted against the N statistic of the assembly [for instance, N40 (x-axis) is equal to about 1 Kbp (y-axis), which means that (100−40 = 60) % of the entire assembly is contained in contigs no shorter than 1 Kbp]. Due to frameshifts caused primarily by homopolymer-associated errors in the derived consensus sequence of the contigs, genes from Roche 454 assembly had fewer complete matches in the NR database relatively to their Illumina counterparts (*inset*; results are based on a total of 72,709 gene sequences annotated on contigs that were shared between the two assemblies and were longer than 500 bp).

We obtained (after trimming) a total of 502 Mbp (∼450 bp long reads) and 2,460 Mbp (100 bp pair-ended reads) from Roche 454 and Illumina sequencing, respectively, of the same community DNA sample. For convenience, we called the two sequence data sets Lanier.454 and Lanier.Illumina, respectively. The sample comprised DNA from the prokaryotic fraction of a planktonic microbial community of a temperate freshwater lake (Lake Lanier, Atlanta, GA); the complexity of the community sampled (in terms of species richness and evenness) was estimated to be comparable to that of surface oceanic communities, but lower than that of soil communities [17]. We applied widely used protocols to assemble both sets of reads (see Materials and Methods for details), which substantially collapsed the Lanier.Illumina dataset into 57 Mbp of total unique sequences and the Lanier.454 dataset into 46 Mbp (Fig. 1C); 57.7% and 49.5% of the total reads in the Lanier.Illumina and Lanier.454 datasets, respectively, were singletons (i.e., remained unassembled). Total unique sequences in this case included only contigs longer than 500 bp because shorter contigs were usually characterized by low coverage and thus, were error-prone (Fig. 2A, inset; and in [18]). We found that about 90% of the Roche 454 unique contig sequences overlapped with Illumina contig sequences (Fig. 1C). It is possible that the remaining ∼10% of the contig sequences might have been different because of imperfect or uneven splitting of the original DNA sample into the two aliquots sequenced and the fact that the diversity in the sample was not saturated by sequencing (estimates based on rarefaction curves using raw reads indicated that we sampled about 80–85% of the total diversity in the Illumina data). Consistent with the results from assembled contigs, we obtained ∼90% of overlapping sequences (∼80% when the overlapping sequences were expressed as a fraction of the total Illumina dataset) between the two datasets when we performed a similar analysis using all raw (not assembled) reads (Fig. 1A). These results revealed that, in general, the two platforms sampled the same fraction of the total diversity in the sample. We also estimated the abundance of each contig shared between the two assemblies by counting the number of reads composing the contig, which can be taken as a proxy of the abundance of the corresponding DNA sequence in the sample [19]. We found a strong linear correlation (*r^2^*>0.99) between the Roche 454 and Illumina data with this respect (Fig. 1D). Therefore, the two platforms provided comparable *in situ* abundances for the same genes or genomes.

Illumina-specific unique contig sequences (16 Mbp) were more than three times as many as the Roche 454-specific ones (5 Mbp), and these additional contigs were attributed to the larger Illumina dataset rather than sequencing artifacts or errors. As evidence of this, analysis of the assemblies of isolate genomes that were sequenced using both platforms (see below) revealed that the extent of chimeric contigs, i.e., contigs that contained contaminating or *in vitro* generated sequences, in the Illumina and Roche 454 assemblies was, on average, less than 0.2% of the total length of the assembled contigs. Although low coverage contigs (e.g., 1 to 5×) are likely to contain a higher fraction of chimeric sequences than 0.2% according to our previous study [18], such contigs were rare in the results reported here, which included only contigs longer than 500 bp with average coverage 10× or higher (only about 3% of the contigs showed less than 5× coverage; Fig. 2A, inset). Lanier.Illumina contigs were generally longer than Lanier.Roche 454 contigs, i.e., the assembly N50 (the contig length for which 50% of the entire assembly is contained in contigs no shorter than this length) was 1.6 Kbp versus 1.2 Kbp, respectively. Even when only a fraction of the total Illumina dataset was used in the analysis that was comparable to the size of the Roche 454 dataset (i.e., 500 Mbp), the derived Illumina assemblies were similar to those of Roche 454 (N50 values were 990 bp for Illumina and 1193 bp for Roche 454; Fig. 2B).

### Sequencing errors in assembled contigs

We evaluated the type and frequency of errors in assembled contigs from metagenomic data using both a comparative and a reference genome approach. In the former approach, we examined protein-coding sequences recovered in contigs longer than 500 bp that were shared between the Lanier.454 and Lanier.Illumina assemblies. We identified 0.4 million homopolymers (three identical consecutive nucleotide bases or more), of which 14 thousand (3.3% of the total) disagreed on length between the two assemblies, resulting in alternative amino acid sequences for about 7% of the total 72,709 gene sequences evaluated. Among these genes, Roche 454 data appeared to have the wrong (artificial) sequence more often than Illumina data. For instance, searching all genes shared between the two assemblies against NCBI's Non Redundant (NR) protein database (Blastx) returned more complete matches with the Lanier.Illumina than the Lanier.454 data, regardless of the identity and e-value threshold used (14% more on average; Fig. 2B, inset). These results were attributable to a higher number of (artificial) frameshifts, caused by homopolymer-associated base call errors, present in the Lanier.454 versus the Lanier.Illumina assembled sequences.

**Figure 3:**
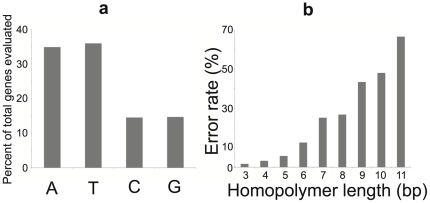
Characteristics of homopolymer-related sequence errors in Roche 454 metagenome assembly. (**A**) A's and T's contribute significantly more homopolymer errors than C's and G's. The average G+C% content of the metagenome was 47.4%; thus, our results are not simply attributable to higher abundance of A's and T's in the metagenome. (**B**) Error rate (as a percentage of the total genes evaluated, y-axis) increases as homopolymer length increases (x-axis).

**Figure 4:**
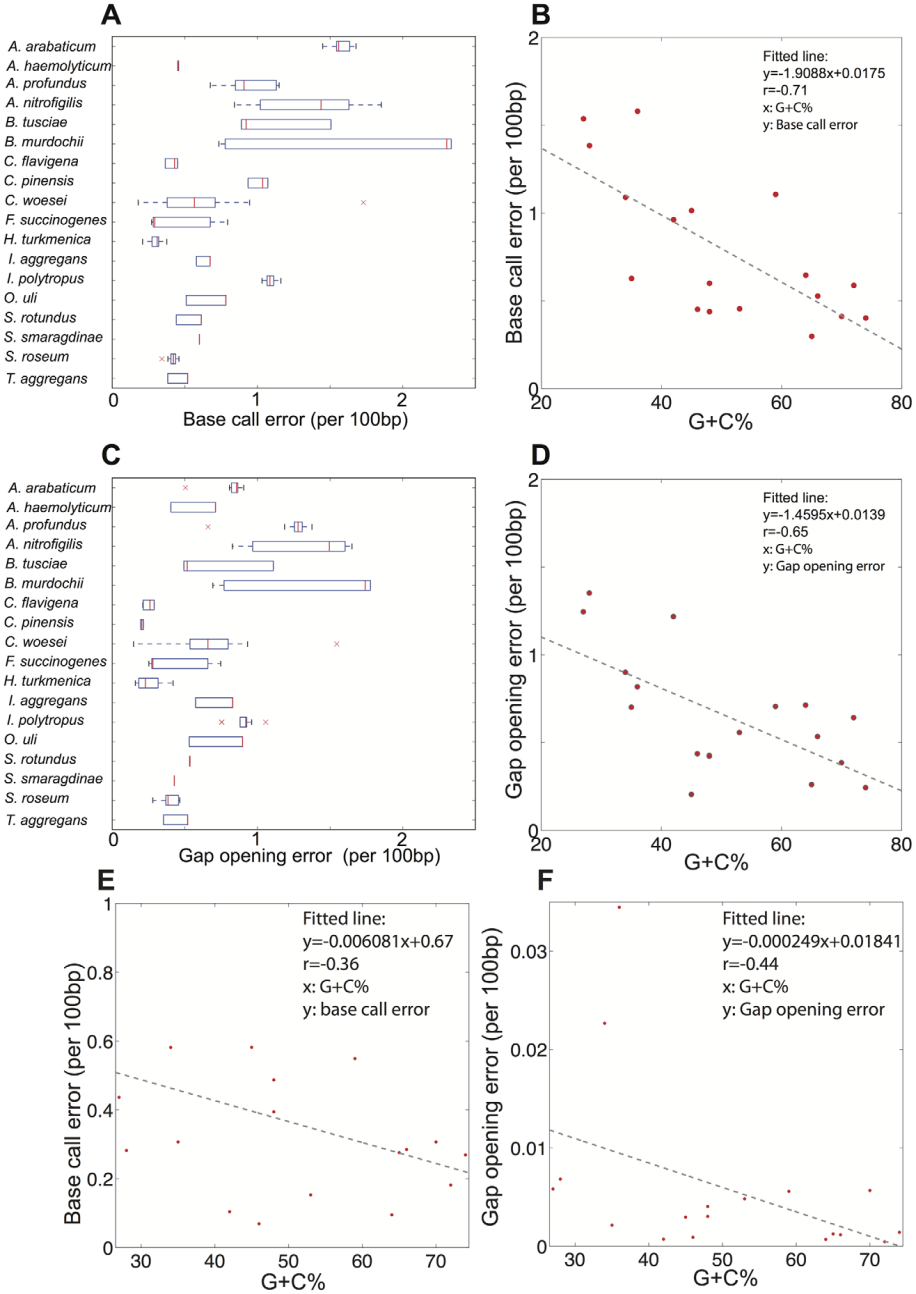
Roche 454 and Illumina GA II read sequence quality based on isolate genome data. Roche 454 sequencing quality is evaluated in panels A through D, which show: (**A**) base call error rate of individual reads (x-axis) for each genome evaluated (y-axis); (**B**) base call error rate (y-axis) plotted against the G+C% of the genome; (**C**) gap opening error rate of individual reads (x-axis) for each genome evaluated (y-axis); (**D**) gap opening error rate (y-axis) plotted against the G+C% of the genome. Illumina GA II sequencing quality is evaluated in panels E and F, which show: (**E**) base call error rate of individual reads plotted against the G+C% of the genome; and (**F**) gap opening error rate of individual reads plotted against the G+C% of the genome. Panels A and C represent the variation observed in reads from different (replicate) datasets of the same genome; red bars represent the median, the upper and lower box boundaries represent the upper and lower quartiles, and the upper and lower whiskers represent the largest and smallest observations. All 2D plots (panels B, D, E, and F) represent the arithmetic average of the medians of each dataset for the same genome; Illumina medians were identical among replicate datasets; therefore, only one value is shown in panel E. The results show that Illumina sequence quality was affected less than that of Roche 454 by the G+C% content of the sequenced DNA (note the lower r-squared value and the slope in E). Thus, the results reported for Illumina based on the metagenome of Lake Lanier (47 G+C%) should be also applicable to metagenomes with different G+C% contents.

In the reference genome approach, genes annotated in the Lanier.454 and Lanier.Illumina contigs were compared against their orthologs in publicly available genomes, and homopolymer errors were identified assuming the publicly available sequences contained no errors. We found that homopolymer errors affected 2.13–2.78% and 0.32–1.02% of the total genes evaluated for the Lanier.454 and Lanier.Illumina data, respectively (dividing by the average gene length, 950 bp, provided the per base error rate; range was estimated from 100 replicates using Jackknife resampling), despite the fact that sequencing error in the raw reads of the two platforms was comparable (∼0.5% per base, in our hands). These percentages were similar to those reported above based on the comparative method (the 3.3% of homopolymers that disagreed between the two datasets includes both Roche 454- and Illumina-specific homopolymer errors). A closer investigation revealed that Roche 454 homopolymer sequence errors were biased toward A's and T's over C's and G's, and the errors were more frequent in homopolymers of greater length (Fig. 3). These patterns were not as pronounced in the Illumina data, indicating that Illumina errors were (more) randomly distributed than Roche 454 errors (see Fig. 4, which is based on isolate genome data).

Single-base sequencing errors increased by an average of 2% when non-homopolymer-associated errors were also taken into account for both platforms. The frequency of single-base errors decreased with higher coverage of the corresponding contigs, i.e., the frequency dropped by about ten fold in contigs with 20× coverage relative to contigs with 2× coverage, reaching a plateau at about 20× coverage. We did not observed a significant difference in error frequency in contigs with higher than 20× coverage (standards on length and coverage for identifying error-prone Illumina contigs are defined in our previous study [18]). Given that the single-base error of individual reads was comparable between Lanier.454 and Lanier.Illumina (∼0.5% per base), our results reveal that the lower single-base error rate of Lanier.Illumina contigs (∼3% vs. ∼4.5% for Roche 454, counting homopolymer- and non-homopolymer-associated errors) is primarily due to the higher coverage obtained. Consistent with these interpretations, we found that the single-base error of Illumina contigs increased by about 0.07% when we removed reads from the assembly so that the average coverage of the Illumina contigs would approximate the average coverage of the Roche 454 contigs (∼8×). It is, however, currently economically unfavorable to obtain similar coverage with the Roche 454 sequencer to the Illumina data (see Discussion below).

We also found that the systematic single-base errors associated with GGC-motifs in Illumina data reported recently [16] represented only a minor fraction of the non-homopolymer-associated errors (0.015% of the total bases analyzed, consistent with the frequency reported in the original study). Hence, the majority of non-homopolymer-associated errors remain challenging to model and thus, to correct. Finally, gene calling on individual reads (as opposed to assembled contigs) was found to be less error prone in Lanier.454 reads than in Lanier.Illumina reads, mainly due to the longer read length. For instance, protein sequences called on Lanier.454 reads had ∼10% more Blastp matches to reference genes from the Lanier.454 assembly than did protein sequences from Lanier.Illumina reads against the Lanier.Illumina reference assembly (Fig. 1B). Thus, Roche 454 is advantageous with respect to gene calling when working with unassembled reads.

### Analysis on isolate genome data

**Figure 5:**
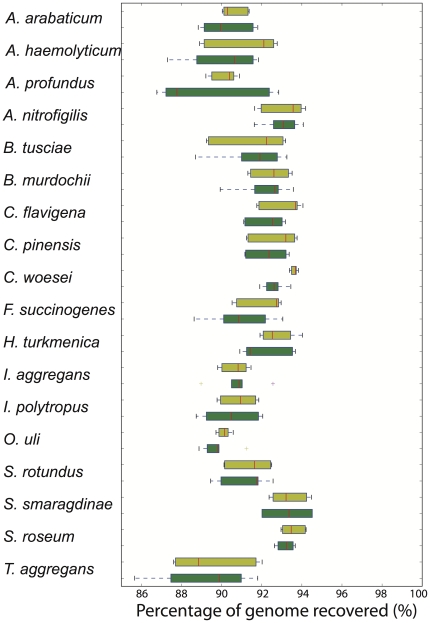
Percentage of reference genome recovered by Illumina (yellow) and Roche 454 (green) assemblies. Graph shows the variation observed in assemblies from different (replicate) datasets of the same genome; red bars represent the median, the upper and lower box boundaries represent the upper and lower quartiles, and the upper and lower whiskers represent the largest and smallest observations. Note that Illumina assemblies recovered a significantly larger fraction of the reference genome than Roche 454 assemblies (two tailed Whitney-Mann U test p-value = 0.014), which is consistent with the results from the metagenomes (Fig. 2). The results for the isolate genomes were based on Illumina input reads that were about 5 times as many as the Roche 454 input reads to provide a ratio that was similar to that of the metagenomic comparisons (5∶1).

To validate our findings from metagenomics, we performed similar comparative analyses based on eighteen isolate genomes that were sequenced by both Illumina and Roche 454 and showed a range of genome sizes and G+C% content (Table 1). Consistent with the metagenomic observations, we found that Roche 454 assemblies from genome data contained a significantly higher portion of frameshift errors compared to Illumina assemblies from the same genome, when the assemblies were built with 5 times more Illumina data than the Roche 454 data, matching the relative ratio of the metagenomic data reported above. Specifically, in genomes of about 50% G+C content (similar to the 47% G+C of the Lake Lanier metagenome), Roche 454 assemblies showed about 5% more frameshift errors than those of Illumina assemblies. This corroborated our estimated error rate in metagenomic data, i.e., that the Lanier.454 assembly had 7% more frameshift sequences than the Lanier.Illumina assembly (Fig. 2). Noticeably, due to the inherent biases of the Roche 454 sequencing approach to produce more frameshifts in A and T rich DNA (Fig. 3), low G+C% genomes sequenced with this platform may have 20% or more genes with frameshift errors whereas the Illumina platform is not affected as much by the G+C% of the sequenced DNA (Fig. 4). These findings call for special attention in cases where the sequenced DNA (e.g., community or isolate genome) is of low G+C%. Further, the single-base sequence and gap opening error rates of individual reads were typically higher by 0.5% and a factor of 10, respectively, for the Roche 454 compared to the Illumina reads (Fig. 4), despite the fact that reads were trimmed based on the same quality standard prior to the analysis. As noted above, similar gap opening errors were observed for the metagenomic reads from the two platforms and single-base accuracy was comparable between the two platforms (99.34% vs. 99.46% for the Lanier.454 and Lanier.Illumina metagenomic reads, respectively). The slightly higher single-base accuracy of Roche 454 metagenomic reads relative to that of the isolate genome reads is presumably due to the use of the latest, optimized Roche 454 protocol in the former and slight differences in the performance of the sequencers used. Finally, in all genomes analyzed, Illumina assemblies consistently recovered a larger percentage of the reference genome than Roche 454 assemblies (two tailed Whitney-Mann U test *p-value* = 0.014; Fig. 5), which was consistent with our observations on the assembly N50 values of the metagenomes (Fig. 2).

**Table 1:** Isolate genomes used in the analysis.

Species	RefSeq	Genome size (Mb)	GC (%)	% coding	Protein coding genes	Size of 454 data (Mb)	Size of Illumina data (Mb)
*Acetohalobium arabaticum* DSM 5501	NC_014378	2.47	36	85	2,282	603	2,982
*Arcanobacterium haemolyticum* DSM 20595	NC_014248	1.99	53	86	1,731	252	2,871
*Archaeoglobus profundus* DSM 5631	NC_013741	1.56	42	91	1,819	600	4,479
*Arcobacter nitrofigilis* DSM 7299	NC_014166	3.19	28	92	3,126	504	6,087
*Bacillus tusciae* DSM 2912	NC_014098	3.38	59	84	3,150	124	2,285
*Brachyspira murdochii* DSM 12563	NC_014150	3.24	27	85	2,809	331	5,115
*Cellulomona flavigena* DSM 20109	NC_014151	4.12	74	90	3,678	563	3,394
*Chitinophaga pinensis* DSM 2588	NC_013132	9.13	45	88	7,192	161	3,769
*Conexibacter woesei* DSM 14684	NC_013739	6.36	72	93	5,914	303	2,578
*Fibrobacter succinogenes* substr. succinogenes S85	NC_013410	3.84	48	90	3,085	769	3,275
*Haloterrigena turkmenica* DSM 5511	NC_013743	3.89	65	84	3,739	205	2,581
*Ignisphaera aggregans* DSM 17230	NC_014471	1.88	35	86	1,930	258	2,739
*Ilyobacter polytropus* DSM 2926	NC_014632	2.95	34	85	1,889	210	5,854
	NC_014633 (plasmid)	0.96	34	83	992		
*Olsenella uli* DSM 7084	NC_014364	2.05	64	86	1,739	248	3,542
*Segniliparus rotundus* DSM 44985	NC_014168	3.16	66	90	3,006	245	3,170
*Spirochaeta smaragdinae* DSM 11293	NC_014363	4.63	48	92	4,219	509	3,306
*Streptosporangium roseum* DSM 43021	NC_013595	10.34	70	85	8,945	373	2,506
*Thermosphaera aggregans* DSM 11486	NC_014160	1.32	46	90	1,387	243	3,181

**Figure 6:**
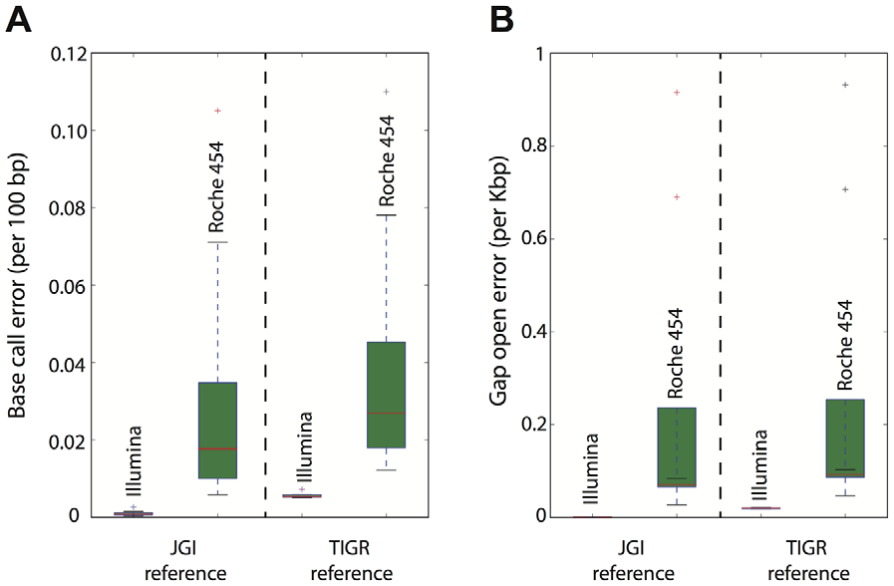
Comparisons of Illumina and Roche 454 assemblies against an independently sequenced reference genome. Nine Illumina and eight Roche 454 assemblies from independent replicate datasets of the *Fibrobacter succinogenes subsp. succinogenes* S85 genome sequenced at JGI were compared against the reference assemblies from the JGI and TIGR genome projects of *Fibrobacter succinogenes subsp. succinogenes* S85. Graphs show the calculated base call error rate (**A**) and gap open error rate (**B**) for each comparison (figure key). Red bars represent the median, the upper and lower box boundaries represent the upper and lower quartiles, and the upper and lower whiskers represent the largest and smallest observations.

**Figure 7:**
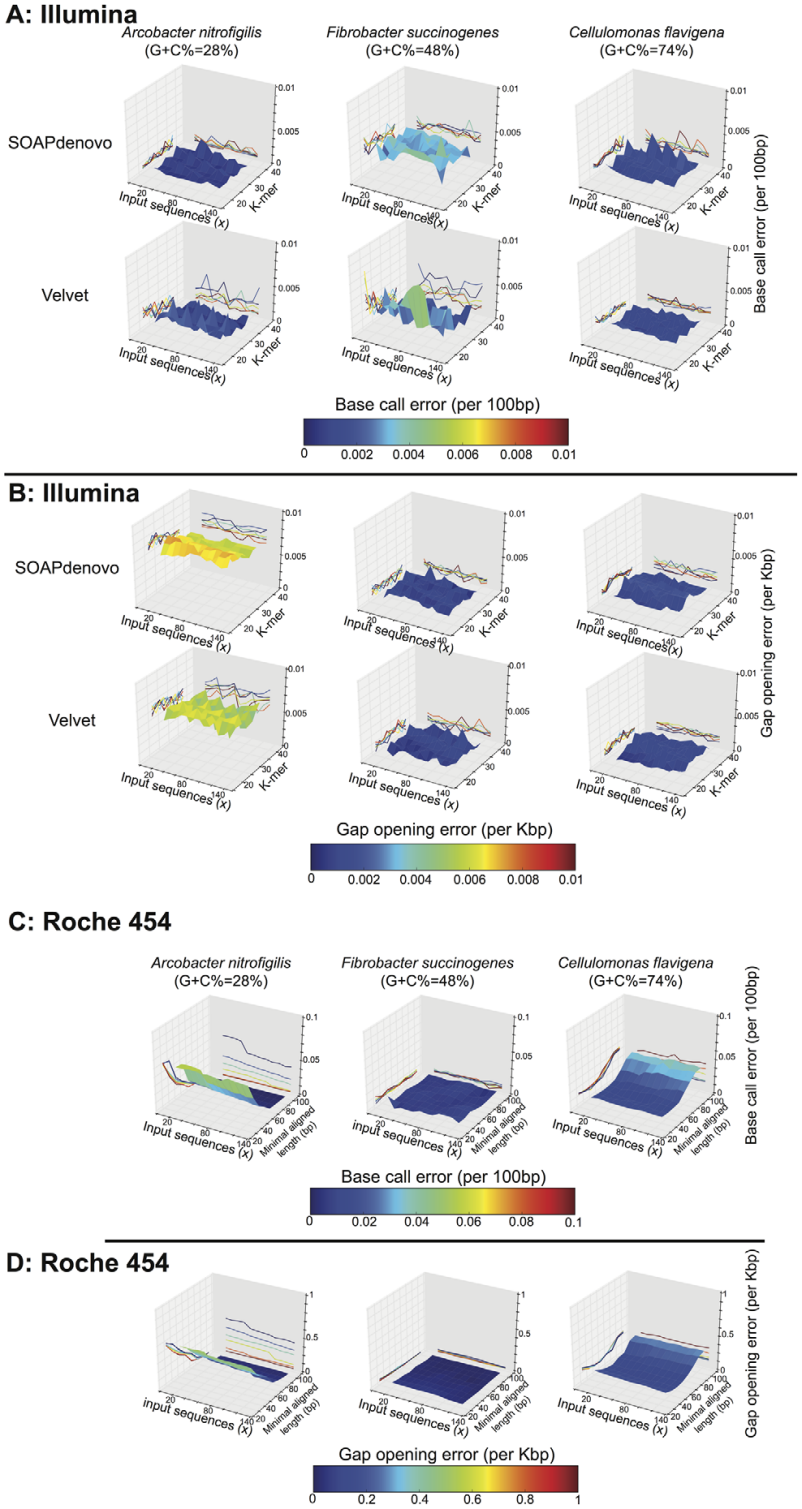
Dependence of the quality of assembled contigs on the parameters of the Illumina assembly. Assembly parameters (primary and secondary x-axes) were evaluated for low (*Arcobacter nitrofigilis*, 28%; left), medium (*Fibrobacter succinogenes*, 48%; middle), and high (*Cellulomonas flavigena*, 74%; right) G+C% genomes. For each genome, a 2D-grid assembly was performed, varying the size of input sequences (20×, 30×, 40×, …, 130×) and the K-mer (21, 23, 25, …, 37) of each of the assemblers used (SOAPdenovo and Velvet). The quality of the resulting contigs was examined in terms of base call error (**A**) and gap opening error (**B**), which revealed that the combination of the parameters of the assembly did not have a dramatic effect on the quality of the contigs (see projected contours on x-z and y-z space). Similarly for the Roche 454 data, a 2D-grid assembly was performed, varying the size of input sequences (20×, 30×, 40×, …, 130×) and the minimal aligned length to merge contigs or reads (30 bp, 40 bp, …, 100 bp) for Newbler. The quality of the resulting contigs was examined in terms of base call error (**C**) and gap opening error (**D**), which revealed that the combination of the parameters of the assembly did not have a dramatic effect on the quality of the contigs except in the extreme values of the minimal aligned length (see projected contours on x-z and y-z space), which were avoided in our direct comparisons of Illumina versus Roche 454 assemblies.

It should be mentioned that the RefSeq reference genome sequences (complete or high draft) used in our reference genome approach to detect errors in assembled contigs or genes were not based on independent Illumina and Roche 454 data, but typically represented the consensus sequence assembled using all Illumina and Roche 454 data available for each genome (hybrid assembly). To eliminate the possibility that our results were biased by the selection of reference genomes, we used the reference assembly of *Fibrobacter succinogenes* subsp. *succinogenes* S85, which was sequenced independently by The Institute for Genomic Research (TIGR GenBank accession: CP002158.1; JGI GenBank accession: CP001792.1). We aligned the assembled contigs from 9 Illumina and 8 Roche 454 assemblies from JGI data for the same genome against the TIGR reference assembly and calculated base call error rate and gap open error rate as described above for JGI genomes. Although the use of the TIGR reference assembly resulted in a slightly higher number of sequence errors for both Illumina and Roche 454 data, Illumina consistently showed a smaller number of sequencing errors and the relative error rate between the two platforms was similar to that based on the JGI genome data alone, independent of the reference genome used (Fig. 6). The higher sequence error rate observed for the TIGR reference genome might be due to the different strain of *F. succinogenes* sequenced or differences in the sequencing platforms or the assembly protocols used by JGI and TIGR. Finally, our evaluations showed that the choices of parameters and amount of input sequence of the assembly did not have any dramatic effect on the quality of the resulting contigs for both Illumina and Roche 454 assemblies (Fig. 7); thus, the assembly step did not substantially affect downstream analyses and our conclusions.

## Discussion

We assessed the advantages and limitations of the Roche 454 and Illumina platforms for metagenomic studies by sequencing the same community DNA sample with each platform. The two platforms agreed on over 90% of the assembled contigs and 89% of the unassembled reads as well as on the estimated gene and genome abundance in the sample (Fig. 1). These findings suggest that both NGS technologies are reliable for quantitatively assessing genetic diversity within natural communities. Moreover, Illumina yielded longer and more accurate contigs (e.g., fewer truncated genes due to frameshifts) despite the substantially shorter read length relatively to Roche 454 and the comparable average sequencing error in the raw reads of the two platforms (∼0.5% per base in our hands; Fig. 2B). In addition, given the monetary savings (e.g., we obtained the Illumina data for about one fourth of the cost of the Roche 454 data), Illumina, and short-read sequencing in general, may be a more appropriate method for metagenomic studies. We also quantitatively assessed the errors in the consensus sequences of the derived assemblies. Roche 454 recovered 14% fewer complete genes than Illumina (Fig. 2B, inset) and this was primarily attributable to a higher sequencing error rate associated with A- and T-rich homopolymers (Fig. 3), which is in agreement with previous results [5], [11]. These errors were not observed in the Illumina data, presumably due to both the high sequence coverage that greatly facilitated the resolution of homopolymer ambiguities and the less pronounced sequencing biases of Illumina (Fig. 4). Nevertheless, about 1% of the total genes recovered in the Illumina assembly contained homopolymer-associated sequencing errors and this number increased to about 3% when non-homopolymer-associated errors were also taken into account (for contigs showing 10× coverage, on average). These results reveal the type and frequency of sequencing errors to expect when performing NGS-enabled metagenomic studies. Although Illumina generally provided equivalent assemblies with Roche 454, there may be cases where Illumina might be inferior to Roche 454. For example, Roche 454 sequencing may be advantageous for resolving sequences with repetitive structures or palindromes or for metagenomic analyses based on unassembled reads, given the substantially longer read length (Fig. 1B).

Although our metagenomic analysis is based on a single community sample, we believe it is robust and informative. Our previous study [17] as well as those of others [20], [21] reported high reproducibility of Illumina-based and 454-based DNA sequencing within the same community sample. More importantly, most of our findings from metagenomic data were reproducible in data from isolate genomes, which were sequenced by both sequencing platforms and showed a range of G+C% content (Figs. 4, 5, 6 and Table 1). Simulations with the isolate genome data also revealed that our conclusions were not substantially affected by the assembly protocols or the amount of input data used (Fig. 7). Some of our results (e.g., assembly N50 comparisons, Fig. 2) should be independent of the NGS platform considered and broadly applicable to short-read sequencing. Lastly, our preliminary evaluation indicates that the latest Illumina sequencer (Hi-Seq 2000) performs similar to Illumina GA-II in terms of read length and quality; hence, our results should be applicable to this sequencer as well.

NGS platforms continue to improve, while new major advancements in sequencing chemistries are on the horizon [22], creating a lot of excitement among microbial ecologists and engineers. The results presented here revealed the errors and limitations as well as the strengths in current metagenomics practice, and should constitute useful guidelines for experimental design and analysis. Our work also provides a methodology for evaluating and comparing metagenomic data from NGS platforms.

## Materials and Methods

### Sampling, DNA extraction, and sequencing

Samples were collected from Lake Lanier, Atlanta, GA, below the Browns Bridge in August 2009 and community DNA was extracted as described previously [17]. The DNA sample was divided into two aliquots of equal volume. One aliquot was sequenced with the Roche 454 FLX Titanium sequencer (average read length, 450 bp) and the other one with the llumina GA II (100×100 bp pair-ended reads) at Emory University Genomics Facility.

### Metagenome assembly and contig error calculation

We obtained a total of 513 Mbp and 3,640 Mbp Roche 454 and Illumina sequence data, respectively. Lanier.454 and Lanier.Illumina reads were trimmed at both the 5′ and 3′ ends using a Phred quality score cutoff of 20. Sequences shorter than 200 bp (Lanier.454) and 50 bp (Lanier.Illumina) after trimming were discarded. The resulting datasets were 502 Mbp (Lanier.454) and 2,460 Mbp (Lanier.Illumina) in size; all our bioinformatic analyses and comparisons were based on these trimmed datasets. Newbler (version 2.0) was used to assemble Lanier.454 with parameters set at 100 bp for overlap length and 95% for nucleotide identity. For Lanier.Illumina, the SOAPdenovo [23] and Velvet [24] *de novo* assemblers were used to pre-assemble short reads into contigs using different K-mers. We performed six independent assemblies, using K = 21, 25, 29 for the three SOAPdenovo runs and K = 23, 27, 31 for the three Velvet runs. The resulting contigs were merged into one dataset, and Newbler was used to assemble this dataset into longer contigs, using the same parameters as in the assembly of Lanier.454 data. Our previous evaluation showed that our hybrid protocol outperforms other approaches for assembling metagenomic and genomic data [18]. Individual reads were mapped against the assembled contigs using Bowtie [25] with default settings to calculate average contig coverage. Protein-coding genes encoded in the assembled contigs were identified by the MetaGene pipeline [26]. Contigs were defined as shared between the assemblies of the Lanier.454 and Lanier.Illumina data when they shared at least 95% nucleotide sequence identity and overlapped by at least 80% of their length (for the shorter contig). The same cut-off was used to map raw reads on contigs. The 95% identity cut-off was used to accommodate the maximum sequencing error observed in raw reads of an isolate genome (about 5%); other cut-offs are not as appropriate as the one used above and were not evaluated.

### Raw (not assembled) read comparisons

We compared the reads from the Lanier.Illumina dataset against the Lanier.454 dataset to identify the fraction of reads shared between the two datasets. Shared reads were defined as those that mapped on reads of the other dataset using Bowtie with default settings [25]. For comparing gene calling accuracy on unassembled reads, we employed FragGeneScan [27] to predict genes on Lanier.454 and Lanier.Illumina reads using the 454 1% error rate model and the Illumina 0.5% error model, respectively. We extracted the predicted gene sequences from the reads and the corresponding amino acid sequences were searched against the genes of the reference assembly of the same dataset using BLAT [28]. The matching gene of the assembly from the protein search using BLAT was compared to the gene matched by the raw read using Bowtie and instances of agreements (matched genes), disagreements (mismatched genes) and “no match found” (BLAT search did not match a gene while Bowtie mapping did) were counted and reported in Fig. 1B.

To estimate the previously described errors associated with GGC motifs in Illumina reads [29], we selected the Roche 454 reads that were covered by at least 10 Illumina reads per base, on average, as reference sequences in Bowtie mapping (∼86.6 Mbp of reads in total). An in-house package written in Python and Perl identified disagreements between Illumina and the reference Roche 454 reads associated with GGC motifs using the rules described previously [29] and counted the number of errors (scripts available upon request).

### Homopolymer error rate

We assessed homopolymer error rate in metagenomic data using two different strategies. First, we examined disagreements in gene sequences annotated on contigs larger than 500 bp and shared between the Lanier.454 and Lanier.Illumian assemblies. For this, Blastn [30] was employed to search all gene sequences annotated in the Lanier.454 assembly against those in the Lanier.Illumina assembly. Reciprocal best matches (RBMs), when overlapping by at least 500 bp and showing higher than 95% nucleotide identity, were identified and re-aligned using ClustalW2 [31]. Homopolymer disagreements between the sequences in the alignment were identified and counted using a custom Perl script (the same approach was applied to the isolate genome data as well). Second, we directly assessed homopolymer error rate against reference genomes from GenBank that represented close relatives (average amino acid identity >70%) of the microorganisms sampled in the Lanier metagenome. To select appropriate genomes, we first identified the putative phylogenetic affiliation of each assembled contig (genus level) in the Lanier.454 and Lanier.Illumina datasets and ranked genera in terms of their abundance. Abundance was determined based on the number and coverage of the contigs, as described elsewhere [17]. Six genomes that represented abundant genera in the lake metagenome were identified this way. The genomes were: Candidatus *Pelagibacter ubique* HTCC1062 (*α-Proteobacteria*), *Opitutus terrae* PB901 (*Verrucomicrobia*), *Polaromonas sp.* JS666 (*β-Proteobacteria*), *Polynucleobacter necessarius* STIR1 (*β-Proteobacteria*), *Synechoccocus sp.* RCC307 (*Cyanobacteria*), and *Synechoccocus sp.* PCC6803 (*Cyanobacteria*). The protein-coding sequences of these genomes were compared against their homologs from the two assemblies to determine homopolymer errors, as described above for direct comparisons between the two assemblies. In order to account for possible biases introduced by uneven genus abundance and provide statistically robust estimates, we employed a Jackknifing resampling method. We sampled 50% of the total homopolymers at random and estimated homolopolymer rate in this subset. The results reported represent averages from 100 iterations. A similar strategy based on reference genome sequences was used to identify and count non-homopolymer-related, single-base errors.

### Analysis of isolate genome data

Assemblies of isolate genome sequences (closed or high-draft) were downloaded from the NCBI RefSeq database (called “reference assemblies” for convenience); raw Illumina and Roche 454 sequencing reads were available through the Joint Genome Institute (JGI, www.jgi.doe.gov). To compare the quality of Illumina vs. Roche 454 contigs assembled from isolate genome data the following approach was followed: Illumina data for each genome was randomly sampled to form several technical replicate datasets, each of which provided about 100× coverage of the reference assembly, on average. Velvet was used to assemble each of these Illumina datasets with K-mer set at 31. Newbler was used to assemble Roche 454 replicate datasets (about 20× coverage on average), using 50 bp minimal alignment length and 95% alignment identity. The amount of Illumina and Roche 454 input sequence data was chosen so that the ratio of the two was similar to the ratio in the metagenomic analysis (2.5 Gb Illumina reads versus 500 Mbp Roche 454 reads, or 5∶1). Between 10 and 15 replicate datasets for each genome and each sequencing platform were analyzed; the exact number depended on the amount of total data available for each genome. Gene sequences from assembled contigs were extracted and ClustalW2 [31] was used to align the sequences against their orthologs from the reference assembly. The alignments were used to count frameshift errors separately for each Illumina or Roche 454 dataset. We also measured the percent of the reference genome recovered in each assembly and the degree of chimerism of contigs as follows: A 500 bp window was used to slide through all assembled contig sequences longer than 500 bp with a step of 100 bp. This resulted in a set of 500 bp long sequence fragments, which were subsequently mapped onto the reference assembly using Blastn. The percent of the reference genome recovered by these fragments as a fraction of the total length of the reference assembly was calculated using a custom Perl script. Similarly, the reference assembly sequence was cut into 500 bp long fragments and mapped onto assembled contigs longer than 500 bp; the unmapped regions of these contigs were identified as chimeric sequences and their total length (as a fraction of the total length of the contigs) represented the degree of chimerism for each dataset. Finally, we calculated the average single-base call error rate and gap opening error rate of individual reads of each dataset as follows: raw reads were trimmed using the same standards as described above and subsequently mapped onto the corresponding reference assembly from RefSeq. Base call errors and gap opening errors were identified as discrepancies between the read sequence and the reference assembly sequence using a custom Perl script.

### Assessing the effect of assembly parameters

We used the isolate genome data to evaluate the effect of the parameters of the assembly on the quality of the contigs as follows: a series of assemblies were obtained for genomes of low (*Arcobacter nitrofigilis*, 28%), medium (*Fibrobacter succinogenes*, 48%), and high (*Cellulomonas flavigena*, 74%) G+C% content. For each genome, we varied the amount of sequences input to the assembly and the primary parameters of assembly (K-mer for SOAPdenovo and Velvet, and minimal alignment length for Newbler). Assemblies were obtained for each possible combination and the base call error and gap opening error of the resulting assemblies were determined as described for individual reads above.
