# Correction: Direct Comparisons of Illumina vs. Roche 454 Sequencing Technologies on the Same Microbial Community DNA Sample

**DOI:** 10.1371/annotation/64ba358f-a483-46c2-b224-eaa5b9a33939

**Published:** 2012-03-29

**Authors:** Chengwei Luo, Despina Tsementzi, Nikos Kyrpides, Timothy Read, Konstantinos T. Konstantinidis

There was an error in the funding statement. The correct funding statement is: This research was supported, in part, by the U.S. Department of Energy (award DE-SC0004601). The Emory Genome Center acknowledges the Georgia Research Alliance and the Atlanta Clinical and Translational Sciences Institute for funding for major equipment purchases. DT acknowledges the support of the Onassis Scholarship Foundation. The work conducted by the U.S. Department of Energy Joint Genome Institute is supported by the Office of Science of the U.S. Department of Energy under Contract No. DE-AC02-05CH11231. No additional external funding was received for this study. The sponsors of this research had no role in study design, data collection and analysis, decision to publish, or preparation of the manuscript. 

